# Advances in Hybrid Fabrication toward Hierarchical Tissue Constructs

**DOI:** 10.1002/advs.201902953

**Published:** 2020-04-07

**Authors:** Paul D. Dalton, Tim B. F. Woodfield, Vladimir Mironov, Jürgen Groll

**Affiliations:** ^1^ Department of Functional Materials in Medicine and Dentistry and Bavarian Polymer Institute University of Würzburg Würzburg 97070 Germany; ^2^ Christchurch Regenerative Medicine and Tissue Engineering (CReaTE) Group Department of Orthopaedic Surgery and Musculoskeletal Medicine Centre for Bioengineering & Nanomedicine University of Otago Christchurch Christchurch 8011 New Zealand; ^3^ New Zealand Medical Technologies Centre of Research Excellence (MedTech CoRE) Auckland 0600‐2699 New Zealand; ^4^ 3D Bioprinting Solutions Moscow 115409 Russia; ^5^ Institute for Regenerative Medicine Sechenov Medical University Moscow 119992 Russia

**Keywords:** biofabrication, biomaterials, manufacturing, organoids, scaffolds, spheroids, tissue engineered medical products

## Abstract

The diversity of manufacturing processes used to fabricate 3D implants, scaffolds, and tissue constructs is continuously increasing. This growing number of different applicable fabrication technologies include electrospinning, melt electrowriting, volumetric‐, extrusion‐, and laser‐based bioprinting, the Kenzan method, and magnetic and acoustic levitational bioassembly, to name a few. Each of these fabrication technologies feature specific advantages and limitations, so that a combination of different approaches opens new and otherwise unreachable opportunities for the fabrication of hierarchical cell–material constructs. Ongoing challenges such as vascularization, limited volume, and repeatability of tissue constructs at the resolution required to mimic natural tissue is most likely greater than what one manufacturing technology can overcome. Therefore, the combination of at least two different manufacturing technologies is seen as a clear and necessary emerging trend, especially within biofabrication. This hybrid approach allows more complex mechanics and discrete biomimetic structures to address mechanotransduction and chemotactic/haptotactic cues. Pioneering milestone papers in hybrid fabrication for biomedical purposes are presented and recent trends toward future manufacturing platforms are analyzed.

## Graphical abstract

There is a growing number of fabrication technologies available, including those based on additive manufacturing principles. Each technology has specific advantages and limitations, and combining them can open new opportunities for the fabrication of complex, hierarchical constructs. Milestone papers in hybrid fabrication for biomedical purposes are identified and trends toward future hybrid biomanufacturing platforms are analyzed.

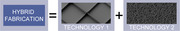

## 1 Introduction

Using automatized technologies for the fabrication of hierarchical structures with the aim to obtain biologically functional tissue analogues has been an essential development within biofabrication.^[^ ^1^, ^2^ ^]^ Such hierarchical 3D tissue constructs have broad applicability including for drug screening,^[^ ^3^ ^]^ disease modeling,^[^ ^4^ ^]^ and organotypic models^[^ ^5^ ^]^ in addition to basic biomedical science research^[^ ^6^ ^]^ and in vivo implantation.^[^ ^7^ ^]^

The central hypothesis of biofabrication is that defined positioning of cells in a 3D hierarchical manner aids the formation of complex tissue.^[^ ^2^ ^]^ This includes “on‐a‐chip” technologies^[^ ^8^ ^]^ where complexity is needed to replicate the cell microenvironment and communication pathways more reflective of the in vivo environment. Automated 3D positioning is therefore expected to improve tissue maturation into more complex, functional ones. While developments in additive manufacturing (AM) technologies have driven much biofabrication research, other fabrication technologies such as electrospinning,^[^ ^9^ ^]^ centrifugal spinning,^[^ ^10^ ^]^ liquid–liquid centrifugal casting,^[^ ^11^ ^]^ uniaxial freezing,^[^ ^12^ ^]^ micromolding,^[^ ^13^ ^]^ and electrochemical compaction^[^ ^14^ ^]^ provide diverse manufacturing options for biomedical researchers. These approaches are also becoming increasingly automated and, as outlined in this review, are part of a greater trend of manufacturing technology hybridization for the creation of complex, hierarchical tissue constructs for biomedical applications.

There are many different automated processes—not only those based on AM principles—available to manufacture 3D tissue constructs.^[^ ^1^, ^15^ ^]^ However, much of the published research for tissue engineering and regenerative medicine (TERM) relies on a single fabrication technology that is expected to achieve all the complex elements required to produce a tissue construct.^[^ ^16^ ^]^ In reality, each manufacturing process has its strengths and weaknesses, with respect to resolution, fabrication rates and the compatibility with cell‐based maturation.^[^ ^1^ ^]^ There are also an exclusive materials or bioink library of each of these fabrication methods.

Ultimately, the complexity and resolution required to mimic natural tissue is greater than what a single technology can deliver. While extracellular matrix (ECM) mimicry is relevant, especially in connective tissue, fabricating a nano‐microhierarchical morphology at the first stage may not be required. While indeed natural tissues have nanoscale elements, there is an abundance of research^[^ ^17^, ^18^, ^19^ ^]^ showing that microscale structures allow cells to attach and then reorganize to produce nanostructured ECM (in other words, a nanoscale scaffold structure or substrate topography is not necessarily required for cells to secrete their natural ECM components). From this perspective, the aim of bioprinting and biofabrication is not maximal ECM mimicry, but final tissue formation and maturation, with ECM ideally secreted and shaped by the cells themselves. Nanoscale mimicry while essential within the mature tissue construct, diverges from what we originally wanted to address in the review, which is hybrid fabrication technologies. Therefore, the hybridization of fabrication technologies from both AM and non‐AM origins is the next logical step. When combining complementing manufacturing technologies, it is possible that the limitations of each individual one can be mitigated.

**Figure 1:**
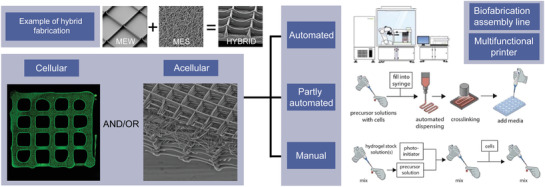
A graphical depiction of melt electrowriting (MEW) and melt electrospinning (MES) as an example of hybrid fabrication (top) and an overview how such technologies can be combined within a biofabrication or tissue engineering and regenerative medicine (TERM) paradigm. Hybrid fabrication approaches are influenced by the level of automation, with a fully automated scenario envisioned in the future. Example inset images previously unpublished and provided by Mr. Marius Berthel. Cellular figure reproduced with permission.^[^ ^31^ ^]^ Copyright 2017, American Chemical Society. Acellular figure reproduced with permission.^[^ ^32^ ^]^ Copyright 2018, The Authors, Published by WILEY‐VCH Verlag GmbH & Co. KGaA, Weinheim. Automated to manual right‐hand side images reproduced with permission.^[^ ^33^ ^]^ Copyright 2019, IOP Publishing.

There is already a trend toward hybridization of automated technologies,^[^ ^20^, ^21^, ^22^, ^23^ ^]^ which we believe will increase over the next decade. Since the term “hybrid” has different meanings depending on the context, this article uses the word to describe the combination of individual manufacturing technologies (simultaneously or in series) to provide a multimodal,^[^ ^24^, ^25^ ^]^ multiphasic,^[^ ^26^, ^27^ ^]^ or multimaterial structure^[^ ^7^, ^28^ ^]^ from which to support tissue maturation or regeneration. As depicted in **Figure** **1**, these hierarchical structures can be grouped as acellular and cell‐based technologies; the first instance involves the fabrication of acellular biomaterials and scaffolds/lattices, while the latter involves cellular incorporation as an integral part of the fabrication technology. For reproducibility reasons, we foresee manual aspects of fabrication such as pipetting and transfer of substrates to eventually become fully automated technologies based on robotization.

## 2 Hybrid Fabrication: Specific Case Analyses and Emerging Key Technologies

**Figure 2:**
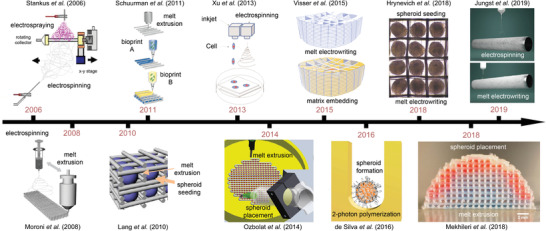
Timeline and milestone papers toward hybrid fabrication for TERM and biofabrication. Schematic of Stankus et al. reproduced with permission.^[^ ^39^ ^]^ Copyright 2006, Elsevier. Schematic of Schuurman et al. reproduced with permission.^[^ ^40^ ^]^ Copyright 2013, IOP Publishing. Schematic of Xu et al. reproduced with permission.^[^ ^41^ ^]^ Copyright 2013, IOP Publishing. Schematic of Visser et al. reproduced with permission.^[^ ^42^ ^]^ Copyright 2015, The Authors. Published by Springer‐Nature Publishing. Figure of Hrynevich et al. reproduced with permission.^[^ ^32^ ^]^ Copyright 2018, The Authors, Published by WILEY‐VCH Verlag GmbH & Co. KGaA, Weinheim. Schematic of Jungst et al. reproduced with permission.^[^ ^21^ ^]^ Copyright 2019, The Authors, Published by Wiley. Schematic of Moroni et al. reproduced with permission.^[^ ^43^ ^]^ Copyright 2008, Wiley. Schematic of Lang et al. reproduced with permission.^[^ ^44^ ^]^ Copyright 2010, IEEE. Schematic of Ozbolat et al. reproduced with permission.^[^ ^45^ ^]^ Copyright 2014, Elsevier. Schematic of Silva et al. reproduced under the terms and conditions of the Creative Commons CC BY 3.0 License.^[^ ^46^ ^]^ Copyright 2016, The Authors, Published by PLOS ONE. Figure of Mekhileri et al. reproduced under the terms and conditions of the Creative Commons CC BY 3.0 License.^[^ ^20^ ^]^ Copyright 2018, The Authors. Published by IOP Publishing.

There are several milestone and breakthrough articles highlighted in **Figure** **2**; studies that first describe and demonstrate the convergence of different fabrication technologies to manufacture more complex 3D tissue constructs. Advantages and disadvantages of specific fabrication technologies used for TERM are summarized in **Table** **1**. All of the following technologies are envisioned to eventually be incorporated into a fully automated biofabrication system as shown in Figure 1. This hybrid fabrication would likely be part of a greater future automated platform based on high‐throughput screening and computational modeling.^[^ ^33^ ^]^

**Table 1:** Comparative advantages and disadvantages of different selected fabrication technologies used within TERM and biofabrication

Technology 1 (T_1_)	Technology 2 (T_2_)	Advantages	Disadvantages	Refs.
Solution Electrospinning	Electrospraying	Low cost; simple to establish;	Lack of polymer/solvent options; generation of airborne particles;	^[^ ^39^ ^]^
Solution Electrospinning	Extrusion‐based Printing	Low cost; high total volume; surface area; improved in vitro	Solvent use; volume fraction occupied by polymer high;	^[^ ^43^, ^48^, ^49^, ^50^ ^]^
Solution Electrospinning	Inkjet	Cell deposition control;	Solvent use; electrospun substrate required on fluid; small pore size;	^[^ ^41^ ^]^
Extrusion‐based Printing	Extrusion‐based Bioprinting	High total volume;	Volume fraction occupied by polymer high;	^[^ ^40^, ^54^ ^]^
2PP	Spheroids	Individual building blocks have mm sizes;	High establishment cost; requires better seeding efficiency;	^[^ ^46^ ^]^
Extrusion‐based Printing	Spheroids	Dispensing control; mm‐sized cell building blocks;	Complex printer; volume fraction occupied by polymer high;	^[^ ^20^, ^70^ ^]^
MEW	Spheroids	Low polymer fraction; design variations;	Time to manufacture; manual seeding;	^[^ ^32^, ^81^ ^]^
MEW	Extrusion‐based Bioprinting	Low polymer fraction reinforcement;	Commercial or custom‐built printer required;	^[^ ^97^ ^]^
MEW	Electrospinning	Reinforcement of solution electrospun tube with minimal layers; MEW layers align cells;	Custom‐built printer required;	^[^ ^21^ ^]^
Melt Electrospinning	Electrospraying	Decoupling scaffold mechanics from level of drug delivery;	Airborne generation of small particles; ventilation and safety required for electrospraying;	^[^ ^100^ ^]^

### 2.1 Solution Electrospinning

Undoubtedly, solution electrospinning^[^ ^34^ ^]^ has had a profound impact on TERM research. Well‐reviewed in depth elsewhere, electrospinning is a processing method that stretches charge polymer solutions/melts into nano‐ and ultra‐fine fibers.^[^ ^35^, ^36^ ^]^ Solution electrospinning is particularly pertinent for tissue engineering, as first illustrated in 2002 when Bowlin and colleagues electrospun collagen fibers.^[^ ^37^ ^]^ This created significant interest in using electrospinning to mimic collagen fibrils, however the solid nature of collectors tended to result in compact fibrous nonwoven sheets with minimal porosity for cell penetration.^[^ ^38^ ^]^

In 2006, Stankus et al. circumvented this challenge of cell penetration into solution electrospun meshes by simultaneously solution electrospinning and cell electrospraying^[^ ^39^ ^]^ (Figure 2). Since most volatile solvents are toxic, and water can be slow to evaporate, this study used hexafluoroisopropanol (HFIP) to dissolve the elastomeric poly(ester urethane)urea into an electrospinning solution while smooth muscle cells isolated from the rat aorta were suspended within Dulbecco's Modified Eagle Medium and electrosprayed onto either a flat collector and a tubular mandrel. One benefit of a tubular mandrel in this instance is that the positions of the two nozzles/spinnerets could be positioned diametrically opposite to each other—this configuration is one repeated in numerous other papers involving tubular collectors. The cells/fibers formed striated layers even though deposition of the two sources was simultaneous. In 2008, Ekaputra et al. simultaneously combined electrospraying of a osteoblast‐containing heparin/hyaluronic acid matrix with solution electrospun PCL fibers and showed that leachable PEO fibers only minimally improved scaffold porosity in this architecture.^[^ ^47^ ^]^

**Figure 3:**
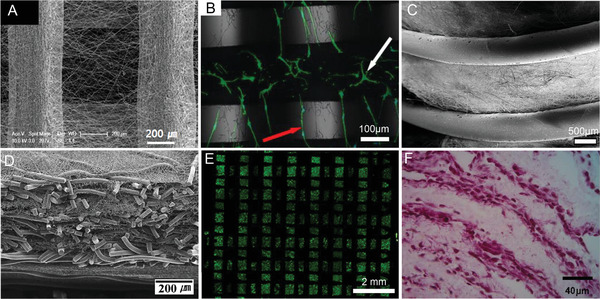
Hybrid fabrication technologies. Combination of material extrusion and electrospinning on A) flat surfaces and B) seeded with MC3T3 cells on a hybrid material‐extruded and electrospun scaffold, shown with white and red arrows respectively. C) A similar approach but with a cylindrical collector. D) Combination of melt electrospinning and solution electrospinning. E) Live/dead staining from a combination of melt‐extrusion and extrusion‐based 3D bioprinting of decellularized bioink containing human inferior turbinate‐tissue‐derived mesenchymal stromal cells. F) Tissue section showing the combination of solution electrospinning and inkjet printing of cells. (A) reproduced with permission.^[^ ^48^ ^]^ Copyright 2008, Elsevier. (B) reproduced under the terms and conditions of the Creative Commons CC BY 3.0 License.^[^ ^49^ ^]^ Copyright 2018, The Authors. Published by MPDI Publishing. (C) reproduced with permission.^[^ ^50^ ^]^ Copyright 2010, IOP Publishing. (D) reproduced with permission.^[^ ^51^ ^]^ Copyright 2010, Elsevier. (E) reproduced.^[^ ^52^ ^]^ Copyright 2018, The Authors. Published by Springer‐Nature Publishing. (F) reproduced with permission.^[^ ^41^ ^]^ Copyright 2013, IOP Publishing.

A different approach to increasing the porosity of electrospun materials also addressed a disadvantage for extrusion‐based fabrication technologies, namely cell seeding efficiency. First to report from two almost simultaneously published papers,^[^ ^43^, ^48^ ^]^ Moroni and colleagues combined extrusion‐based fabrication of molten poly(ethylene oxide‐terephthalate)/poly(butylene terephthalate) with the solution electrospinning of the same, dissolved polymer.^[^ ^43^ ^]^ After seeding with chondrocytes, the electrospun containing group had significantly higher cell entrapment, and the glycosaminoglycan/DNA ratio significantly higher after 28 days. Furthermore, the chondrocytes were spread on the extruded scaffold and were rounded when the electrospun fibers were integrated. In a similar experiment with chondrocytes and with similar findings, Park et al. used poly(ε‐caprolactone) for the two components; one extruded as a melt and the other dissolved in HFIP and electrospun between the layers of the extrusion‐based fabrication process^[^ ^48^ ^]^ (**Figure** **3**A). Similar studies by Mota et al. combining these two fabrication technologies to demonstrate the different morphologies that result on the different diameter surfaces when MC3T3 murine preosteoblasts are seeded Figure 3B.^[^ ^49^ ^]^ Centola et al. was the first to generate such multimodal scaffolds (Figure 3C) on tubular collectors, using material extrusion upon solution electrospun membranes.^[^ ^50^ ^]^ Recently, solution electrospun tubes were hybridized with melt electrowriting by Jungst and colleagues, (Figure 2), to make multiphasic tubes from PCL for vascular applications.^[^ ^21^ ^]^

While solution electrospinning attracted extensive attention for tissue engineering, melt electrospinning typically produced larger diameter fibers^[^ ^53^ ^]^ that were (at the time) beyond the nanoscale dimensions that drove nanotechnology research. However, this size discrepancy was used in a hybrid fabrication approach, Kim et al. combined melt electrospinning of poly(lactide‐*co*‐glycolide) (PLGA) to establish large fibers, while dissolving PLGA into HFIP and electrospinning the solution produce the sub‐micrometer diameter fibers (Figure 3D).^[^ ^51^ ^]^ Similar to the aforementioned articles, this configuration used two diametrically opposed nozzles/spinnerets and collected onto a tubular mandrel in the middle. Such a combined structure provides volume due to the larger diameters and enables improved cell seeding with human epidermal keratinocytes.^[^ ^51^ ^]^

### 2.2 Bioprinting Approaches

While electrospinning is often used as a second process to introduce a high surface area for cell attachment,^[^ ^50^ ^]^ the combination of bioprinting with extrusion‐based fabrication technologies was adopted by Malda and colleagues (Figure 2) for a different reason.^[^ ^40^ ^]^ When bioprinting a cell‐laden hydrogel for several layers to achieve volume, the weight of the upper layers of the bioink imparts a significant force on the underlying layers and the structure can collapse or become unstable. Therefore, a more rigid, material extruded (typically a biodegradable thermoplastic polymer such as PCL) fiber is used to minimize the forces imparted on the weaker bioink.^[^ ^40^ ^]^ Decellularized tissue was also used as bioinks by Pati et al. within a material extruded scaffold as shown in Figure 3E.^[^ ^54^ ^]^

In 2013, Xu et al. demonstrated a different hybrid printing approach involved electrospinning and inkjet bioprinting of cells (Figure 2).^[^ ^41^ ^]^ A solution of PCL and Pluronic F‐127 was dissolved in acetone and electrospun onto a phosphate‐buffered saline‐filled Petri dish after which chondrocytes/fibrinogen/collagen were dispensed with an inkjet valve. This process was repeated until there were three electrospun layers and two bioink layers, 1 mm thick. Cell viability in vitro and their evaluation as hybrid cartilage constructs in vivo was performed (Figure 3F). The mechanics of the hybrid constructs were superior compared to the printed hydrogel alone and the chondrocytes remained viable while producing cartilage‐specific extracellular matrix.^[^ ^41^ ^]^

As outlined in‐depth later in this review, the melt electrowriting (MEW) of PCL fibers provided an alternative reinforcement structure for such voluminous hydrogel structures.^[^ ^42^ ^]^ What is distinct about the research reported by Visser et al (Figure 2) is that significant mechanical reinforcement could be obtained under compression with 19 µm diameter MEW filaments that occupied between only 2% and 7% of the total construct volume. Individually, the PCL scaffold and hydrogel had a compressive moduli of ≈10–15 kPa, however substantially increased to 405 kPa when combined.^[^ ^42^ ^]^ Numerous studies on such soft network composites have confirmed that small volume fractions of well‐positioned, small diameter fibers can profoundly affect the mechanical properties.^[^ ^55^, ^56^, ^57^ ^]^ MEW has also recently been performed to generate chemically crosslinked hydrogels for biomedical applications,^[^ ^58^ ^]^ and could be considered a secondary structure similar to extrusion‐based systems previously described by Schuurman et al.^[^ ^40^ ^]^

A prerequisite for combining bioprinting with other fabrication technologies is the precise control over the material properties of the biomaterial inks, and in case it is a formulation of cells and materials, the bioinks.^[^ ^59^ ^]^ Crucial material parameters such as a proper rheological behavior, the corresponding characterization, postfabrication curing and shape fidelity, and most recent material and fabrication developments have recently been reviewed in several comprehensive reviews and are thus not further discussed here.^[^ ^60^, ^61^, ^62^, ^63^, ^64^, ^65^, ^66^ ^]^

### 2.3 Tissue Spheroids Bioassembly

Tissue spheroids are densely packed cell aggregates and the idea to use tissue spheroids as building blocks in biofabrication and bioprinting were introduced almost a decade ago.^[^ ^67^ ^]^ There are numerous advantages of using tissue spheroids in biofabrication as building blocks: i) they are 3D and can have maximal cell density for replication of natural condensation reactions observed in developmental tissue biology and tissue formation; ii) they have a spheroidal shape suitable for bioprocessing; iii) they have intrinsic capacity for tissue fusion or formation of larger size tissue engineered constructs via self‐assembly process; iv) it enables self‐assembly/spheroid formation with multiple cell types, i.e., spheroid coculture to replicate the native condensation niche and cell‐cell communication; v) the diameter of tissue spheroids (usually 250 ± 50 µm, also up to 1 mm in diameter) allows significant (up to 20 times) reduction in the number of fabrication layers and, thus, reduce printing time required) spheroids have been shown to form almost all tissue types.

Tissue fusion is a ubiquitous natural process and occurs during embryonic development.^[^ ^68^ ^]^ In order to create 3D tissue constructs from tissue spheroids they must be positioned so that they can fuse. Over the past decade many hybrid fabrication technologies have involved tissue spheroids. Lang et al. (Figure 2)^[^ ^44^ ^]^ first melt‐extruded a porous thermoplastic scaffold and sequentially assembled tissue spheroids into the pores. Mekhileri et al. used a more advanced automated approach employing a specially designed hybrid 3D bioprinter enabling microfluidic singularization and precision placement and insertion of tissue spheroids into scaffolds designed to mimic mechanical properties of the target surrounding native tissue (Figure 2).^[^ ^20^ ^]^ This provides the capacity for the overall construct to be manufactured using a “modular” 3D bioassembly strategy.^[^ ^69^ ^]^ Unlike dissociated cell seeding that is inefficient in extrusion‐based fabrication structures without an electrospun mesh to entrap individual cells, modular spheroids are of such dimensions that they allow 100% seeding efficiency without the electrospun component combined with precision 3D spatial organization or arrangement of individual tissue modules.

Biofabrication of spheroids embedded within 3D bioprinted bioinks was further demonstrated with a “multiarm bioprinter.”^[^ ^45^ ^]^ In this instance spheroids could be placed discretely and a coaxial nozzle permitted the codelivery of an alginate solution with a calcium chloride solution to facilitate crosslinking. This approach enabled the spheroids to be placed and also fixed into position with a reinforcing structure. While such reinforcing structures are extruded to allow matrix and spheroid handling and incubation, at 400–600 µm in diameter, such filaments occupy a significant volume fraction of the overall tissue construct (below 75 vol% porosity).^[^ ^45^ ^]^

While many biofabrication approaches adopt extrusion‐based principles to deposit biomaterial inks and bioinks, light‐based technologies have a different set of capacities. Widely appreciated as one of the best resolved AM technologies, two‐photon polymerization (2PP) can be used to design a different type of reinforcement construct. One example is the “lockyball,” which are spherical, porous containers that have protruding hooks and structures that interlock.^[^ ^46^ ^]^ Silva et al. showed that cells can be seeded within such lockyballs (Figure 2), that facilitate large volume structures through interaction and assembly of the locking structures.

**Figure 4:**
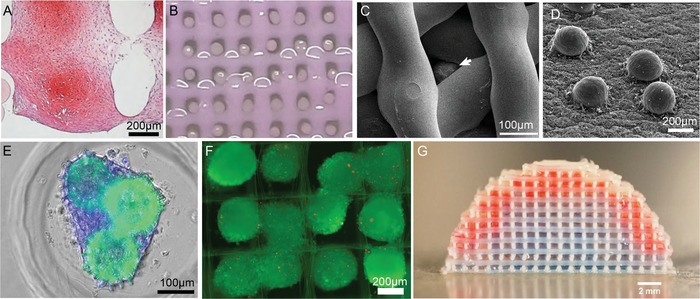
Hybrid biofabrication technologies including manual pipetting (top‐seeding) of 3D tissue. A) Extrusion printing of thermoplastic scaffold combined with precision placement and fusion of tissue spheroids (chondrospheres), stained with Saf‐O. B) Extrusion bioprinting of collagen hydrogel with robotic placement of spheroids. C) Extrusion bioprinting of collagen hydrogel with placing of ovarian follicle (white arrow) using pipetting. D) Electrospinning of polyurethane matrix with robotic placing of tissue spheroids fabricated from human fibroblasts. E) Biofabrication of tissue spheroids inside synthetic microscaffold ("lockyball") fabricated by 2PP. F) Top seeding of tissue spheroids into scaffold fabricated by MEW. G) Precision bioassembly of multicellular tissue spheroids in sequential layers of a mechanically stable and anatomically shaped 3D scaffold. (A) reproduced with permission.^[^ ^70^ ^]^ Copyright 2012, Springer‐Nature Publishing. (B) reproduced with permission.^[^ ^71^ ^]^ Copyright 2019, Springer‐Nature Publishing. (C) reproduced under the terms and conditions of the Creative Commons CC BY 4.0 License.^[^ ^72^ ^]^ Copyright 2017, The Authors. Published by Springer‐Nature Publishing. (D) reproduced under the terms and conditions of the Creative Commons CC BY 4.0 License.^[^ ^30^ ^]^ Copyright 2016, The Authors, Published by Whioce Publishing Pte Ltd. (E) reproduced under the terms and conditions of the Creative Commons CC BY 4.0 License.^[^ ^46^ ^]^ Copyright 2016, The Authors, Published by PLOS ONE. F) reproduced with permission.^[^ ^32^ ^]^ Copyright 2018, The Authors. Published by WILEY‐VCH Verlag GmbH & Co. KGaA, Weinheim. (G) reproduced under the terms and conditions of the Creative Commons CC BY 3.0 License.^[^ ^20^ ^]^ Copyright 2018, The Authors. Published by IOP Publishing.

When discretely dispensing two different types of spheroids into a dome structure made by extrusion‐based printing Mekhileri et al. (Figure 2) provided a demonstration of future spheroid bioassembly for hierarchical structures.^[^ ^20^ ^]^ Using the osteochondral defect as a target tissue, the extrusion of a thermoplastic copolymer was used to fabricate sequential layers, including the anatomic dome shape of the articular cartilage surface. This paper is the first to show discrete automated 3D bioassembly of cellular spheroids and cell‐laden hydrogel spheroids into reinforcing scaffolds, with the automated singularization and assembly of spheroids occurring in a microfluidic aqueous environment, i.e., the nozzle is under the surface of the media when dispensed. This strategy represents an automated layer‐by‐layer hybrid biofabrication approach alternating between extrusion‐based 3D plotting of thermoplastic polymer and microfluidic bioassembly of preformed cellular spheroid modules. This research group also described the development of a 96‐well plate bioreactor showing perfused drug screening on 3D bioassembled cocultured cancer spheroid model (i.e., 3D plotted scaffold + coculture cancer spheroids).^[^ ^73^ ^]^ Similar hybrid approaches have been employed by Ozbolat and colleagues (Figure 2) using alginate hydrogels for fabricating porous scaffolds suitable for placing tissue spheroids.^[^ ^45^ ^]^ 3D Bioprinting Solutions, a company in Russia, also developed a hybrid biofabrication technology using original multifunctional bioprinter “Fabion”^[^ ^74^ ^]^ (**Figure** **4**B).

In another approach, tissue spheroids were robotically placed onto a polyurethane electrospun matrix^[^ ^30^ ^]^ (Figure 4D). These tissue spheroids attach and spread on the surface of an electrospun matrix. Precision robotic placement and patterning of tissue spheroids on the surface of electrospun matrices allowed control over the thickness of resultant 3D tissue construct after attachment, spreading and tissue fusion. Moreover, it has been suggested (but not yet implemented) that tissue spheroids could be also bioassembled on the opposite side of an electrospun matrix enabling more complex layered 3D tissue construct from different types of tissue spheroids. 3D Bioprinting Solutions used robotic 3D Bioprinting of tissue spheroids biofabricated by rounding of mouse embryonic tissue explants on printed collagen scaffolds for biofabrication of first functional and vascularized organ construct—a mouse thyroid gland.^[^ ^75^ ^]^ Implantation of bioprinted mouse thyroid gland construct under kidney capsular into the experimental animal model of hypothyreosis using radiation ablation allowed for restored levels of the thyroid hormone thyroxine (T4).^[^ ^75^ ^]^ The tissue spheroid could be also formed in microscaffolds fabricated by two photon polymerization^[^ ^46^, ^76^, ^77^ ^]^ (Figure 4E). Additionally, the seeding and capture of tissue spheroids into specifically designed MEW scaffolds was readily performed^[^ ^32^ ^]^ (Figure 4F). Thus, different types of solution electrospun, melt electrospun, melt‐extrusion based 3D plotting and two‐photon polymerized scaffolds could be used for development of hybrid fabrication technologies combining tissue spheroid assembly. The automated bioassembly of tissue spheroids using fluidic handling and special hybrid 3D bioprinters^[^ ^20^, ^78^, ^79^ ^]^ has an obvious advantage compared with manual placement in further advancing of tissue spheroids based hybrid biofabrication technologies. This also leads to the development of complex automated 3D in vitro tissue models for medium‐ or high‐throughput screening.

Material‐extruded scaffolds for reinforcement often occupy considerable polymer volume; space where cells could be occupying to regulate their microenvironment until the reinforcing part degrades. Conversely, MEW readily produces scaffolds above 80 vol% accessible to the cells.^[^ ^80^ ^]^ In 2018, Hrynevich et al. used MEW in a manner that can create a spectrum of fiber diameters, with each fiber placed in a specific position.^[^ ^32^ ^]^ This was expanded in a separate paper to show such composites can be sectioned, stained and handled; increasing the number to many hundreds of spheroids was demonstrated into a construct that could be further manipulated (Figure 2).^[^ ^81^ ^]^ The capacity to design reinforcing scaffolds at the low‐micrometer resolution provides an additional tool for future hybrid fabrication approaches.

Aspects of the printing process that could cause the structure of the spheroid to be compromised during and after printing have been raised or addressed in literature that has pioneered spheroid bioassembly approaches. For example Mekhileri et al.^[^ ^20^ ^]^ demonstrated that by adopting a fluidic spheroid singularization and insertion print head strategy, cell viability and spheroid shape was maintained and equivalent to non‐bioassembled control spheroids. Furthermore, assembly of cell‐encapsulated hydrogel beads or spheroids offers additional protection to cells from flow, shear or mechanical stress during assembly. The development of hybrid approaches that aim to combine spheroids and/or organoid tissue modules should be cognizant of the biological demands and fragility of spheroid/organoid manipulation during optimization and automation of biofabrication systems and determination of spheroid viability, composition and integrity must be validated throughout all steps of any bioassembly strategy and form a key part of any proof‐of‐concept validation studies.

### 2.4 The Kenzan Method

**Figure 5:**
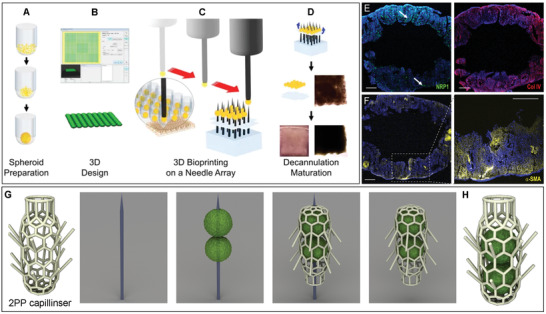
Overview of the Kenzan Method. A–D) Schematic of the needle‐based substrate that allows assembly and consequent fusion of spheroids into a tubular structure. E,F) Immunofluorescent staining of one such construct after one week removed from needles prepared from human induced pluripotent stem cell‐derived smooth‐muscle forming cell spheroids. G,H) A hypothetical hybridization of Kenzan with 2PP, solving a persistent challenge of how to embed spheroids within a capillinser structure. Scale bars for (E,F) are 1 mm. (A–D) reproduced under the terms and conditions of the Creative Commons CC BY 4.0 License.^[^ ^84^ ^]^ Copyright 2017, The Authors. Published by Springer‐Nature Publishing. E,F) reproduced with permission.^[^ ^82^ ^]^ Copyright 2017, Wiley Publishing. G,H) provided by Dr. Fred Pereira.

The Japanese word “Kenzan” originates from a spiky structure used for flower arrangement. From the biomedical fabrication perspective, this approach positions spheroids into 3D arrangements by skewering them onto microneedles (**Figure** **5**A–D) that are spaced sufficiently to allows spheroid fusion. Deformation forces caused by skewering the spheroids^[^ ^82^ ^]^ during assembly are not reported to affect cell viability, ECM production or fusion. With the Kenzan method sheets, tubes and heterogeneous tissue have been fabricated by using the appropriate spheroid types and position.^[^ ^83^ ^]^ For example, Figure 5E,F shows how smooth‐muscle forming spheroids made from human induced pluripotent stem cells form one week after removal from the microneedles. During their positioning on the microneedles, then spheroids fuse and seemed to encapsulate microvascular fragments.^[^ ^82^ ^]^

While the Kenzan method is described as scaffold‐free, one can argue that the microneedles are a “temporal support” that behaves as a scaffold. Irrespective of the definition, it is a fascinating approach that could have particular applicability to hybrid fabrication approaches. Since the Kenzan method allows the physical transfer of tissue structures to another location, they can be sustained for fusion and maturation within a spectrum of environments. One can consider how other AM methods (such as 2PP, MEW; stereolithography (SLA)) can be combined with the Kenzan method to expand the manufacturing possibilities (Figure 5G,H). In this hypothetical example of hybrid fabrication, the challenge of encapsulating spheroids within a 2PP‐fabricated capillinser could be solved using the Kenzan method.

### 2.5 MEW

There are upper and lower diameter limits for electrospinning and microextrusion respectively, due to inherent physical phenomenon with processing such viscoelastic fluids. From a manufacturing perspective, MEW can be considered a hybrid fabrication technology in itself between electrospinning and microextrusion. Distinct from both these two technologies, the fiber diameter is variable, ranging from 800 nm to 150 µm (typically much lower than microextrusion), while fiber placement is excellent (which remains a disadvantage in solution electrospinning). MEW is, unlike bioprinting, a cell‐free manufacturing process, although work on electrohydrodynamic (EHD) jetting with cell‐containing fluids enables this feature.^[^ ^85^, ^86^ ^]^

The EHD jetting of the MEW process relies on a phenomena that stabilizes fluid columns at low flow rates with a voltage that is applied across a nozzle and collector.^[^ ^87^ ^]^ Outlined by Taylor in 1969,^[^ ^88^ ^]^ and shown as “floating water” bridges,^[^ ^89^ ^]^ such applied voltages prevent fluid column break up^[^ ^87^ ^]^ between two points. This differs from electrospinning where a high voltage is used to initiate electrical instabilities (i.e., whipping) in a jet to produce ultra‐fine fibers.^[^ ^35^ ^]^ MEW has been a notable technology for hybrid fabrication, and is reviewed elsewhere in this context.^[^ ^90^ ^]^

**Figure 6:**
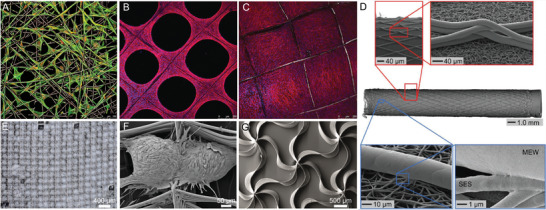
Examples of MEW products and integration within biofabrication. A) Top‐seeded human dermal fibroblasts on a MEW scaffold with a 30˚ pitch deposition. Osteoprogenitor cell line seeded on a 98% porous scaffold after B) six and C) 14 days in vitro, demonstrating typical pore closure for ECM depositing and proliferating cells. D) A multiphasic tube combining a solution electrospun substrate (SES) that is subsequently MEW at a specific angle. E) An adipose‐derived spheroid sheet for transfer, including F) SEM image of two spheroids in adjacent pores. G) sinusoidally printing and intersecting patterns for highly flexible scaffolds or soft network composites. (A) reproduced with permission.^[^ ^91^ ^]^ Copyright 2013, IOP Publishing. (B,C) reproduced with permission.^[^ ^92^ ^]^ Copyright 2015, Mary‐Anne Liebert. (D) reproduced with permission.^[^ ^21^ ^]^ Copyright 2019, The Authors. Published by Wiley. (E,F) reproduced with permission.^[^ ^81^ ^]^ Copyright 2019, The Authors, Published by WILEY‐VCH Verlag GmbH & Co. KGaA, Weinheim. (G) reproduced with permission.^[^ ^55^ ^]^ Copyright 2017, American Chemical Society.

An advantage of MEW is that low‐micrometer‐scale fibers can be accurately placed into position,^[^ ^32^, ^43^, ^48^, ^49^ ^]^ first shown in 2011 by Brown et al.^[^ ^93^ ^]^ MEW scaffolds have been used for both in vitro^[^ ^91^ ^]^ (**Figure** **6**A–C) and in vivo^[^ ^94^ ^]^ research. MEW scaffolds tend to have a high porosity, typically from 80 vol% and even up to 98 vol% pore volume (Figure 6B,C).^[^ ^92^ ^]^ This allows for both cell attachment as well as self‐organization within the scaffold pore. Jungst et al. (Figure 2) combined MEW with solution electrospinning, then seeded with endothelial and stromal cells make tubular vascular grafts. Interestingly, thin solution electrospun PCL layers did not affect the accuracy of MEW PCL fiber placement onto the collector, and good fiber fusion between the two regions was seen (Figure 6D). The capacity to accurately place MEW fibers also leads to their use as a customizable support structure for spheroids (Figure 6E,F).^[^ ^81^ ^]^

The defined placement of MEW fibers has a profound effect on their mechanical properties, when embedded within a second matrix. Bending of MEW fibers is restricted by the matrix and significantly higher mechanical properties can be achieved while maintaining a low volume composite fraction. For example, Visser et al. showed that a MEW fiber‐reinforced matrix was much stronger in compression (≈ 50 times) than the individual components, despite the MEW scaffold occupying only 7% space within the composite volume.^[^ ^42^ ^]^ This form of mechanical reinforcement also extends to shear stresses.^[^ ^95^ ^]^ It is therefore an efficient method to reinforce matrices, including GelMA,^[^ ^42^, ^55^, ^57^ ^]^ alginate,^[^ ^42^ ^]^ and Matrigel.^[^ ^96^ ^]^ Furthermore, the mechanics of the MEW composite can be tuned, by the fiber placement. Direct writing the fibers in a sinusoidal pattern (Figure 5G)^[^ ^55^ ^]^ affects the mechanical properties, with tensile testing demonstrating a distinct “toe region” that can be controlled with different amplitudes and the wavelength laydowns. In another example of MEW used for hybrid fabrication, it was combined with bioprinting to produce a soft network composite.^[^ ^97^ ^]^

The capacity to significantly alter the fiber diameter during printing extends the design capability for MEW scaffolds.^[^ ^32^ ^]^ With an appropriate calibration, MEW scaffolds can be constructed based on their intended dimensions rather than a specific outcome that is usually defined by the stable manufacturing parameters.^[^ ^32^ ^]^ Furthermore, the desired mechanics of a target tissue—demonstrated with heart valves—can be designed into the MEW reinforcing structure so that the reinforced hydrogel displays similar mechanical behavior.^[^ ^98^ ^]^ With comparatively less research performed on this technique compared to electrospinning, the use of such voltage‐stabilized jets offer more options in design. While use of melts is advantageous from a rapid fabrication perspective, the stabilized jet is equally applicable to polymer solutions,^[^ ^99^ ^]^ including bioinks.^[^ ^86^ ^]^ From a fabrication perspective, avoiding the use of organic solvents for MEW has advantages when it comes to potential toxicity and ventilation requirements. However heat can affect chemical stability, and nonwoven fabrics based on melt electrospinning have been previously coupled with solution electrospraying for drug delivery purposes.^[^ ^100^ ^]^

The millimeter‐scale collector distance between the nozzle and the sample in MEW has also enabled monitoring of the jet to obtain useful information. In a research article introduced in 2019 to demonstrate high‐throughput screening of printing parameters (i.e., Printomics), Wunner et al. devised a conveyor‐belt collector system that allowed digitized information to be automatically generated from a fully automated printing system.^[^ ^101^ ^]^ This allows the rapid identification of stable processing within a multiparametric printing process such as MEW. While beyond the scope of this review, such digitized information is well‐suited toward artificial intelligence‐based manufacturing directions. There have been several publications related to machine learning within biofabrication to date.^[^ ^102^, ^103^, ^104^ ^]^

### 2.6 Tomographic Volumetric Bioprinting

**Figure 7:**
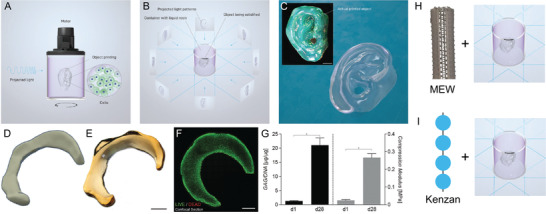
Volumetric bioprinting and its potential utility in hybrid fabrication. A) Schematic showing how incoming light can start a gelation process that B) produces a specific shape in the center when the sample in rotated while the projected image is altered. C) A photograph of the final printed material, which has a smooth surface finish. D) A CAD drawing of a meniscus. E) A photograph of the volumetrically printed product. F) A live/dead stain after 28 days. G) A graph showing significant cell compatibility and improvements in mechanical strength of the tissue. H,I) A demonstration of how hybrid fabrication could be achieved by placing structures within the container prior to volumetric bioprinting. H) A PCL MEW tube with aligned fins that allows light to transmit (or project) into the center. I) A schematic of how spheroids (blue) positioned with the Kenzan method could be further entrapped with a bioresin. (A–I) reproduced with permission.^[^ ^109^ ^]^ Copyright 2019, The Authors, Published by WILEY‐VCH Verlag GmbH & Co. KGaA, Weinheim. (H) includes a photograph of a MEW tube kindly provided by Mr. Thomas Robinson.

Many of the afore‐mentioned fabrication technologies use AM principles, in that the scaffold or construct is made in a layer‐by‐layer approach. Tomographic manufacturing produces objects in a volumetric manner, by projecting a series of images into a rotating container so that an object is only created in the regions where sufficient photopolymerization occurs (**Figure** **7**). Described as being a “reverse tomography” technique, volumetric bioprinting is distinguished by the extremely rapid production of a 3D object—in the range of 20–30 s. This method was inspired by a medical therapy treatment procedure in which a specific dose of ionizing radiation is deposited in 3D by rotating an intensity modulated source around the patient. This was described first by Mackie et al. in 1993.^[^ ^105^ ^]^ The implementation of this technique in the visible light range for photopolymerization was performed independently 25 years later by two groups as described in the works of Kelly et al.^[^ ^106^, ^107^ ^]^ and Loterie et al.^[^ ^108^ ^]^ The latter authors applied this approach to bioprinting, using visible light initiators and incorporated cells within the photopolymerizable resin.^[^ ^109^ ^]^ The current printing resolution for volumetric printing is 80 µm.^[^ ^110^ ^]^

The disruptive potential of volumetric bioprinting on TERM and biofabrication research is substantial, especially if the method can be delivered at an economical price. While the rapid production of millimeter volume cell‐containing constructs is in itself exciting, it is the capability to hybridize volumetric bioprinting with other manufacturing technologies that will drive this technology even further. For example, it is not unreasonable to predict that the photopolymerizable resin can be mixed with other AM fabricated constructs, such as those made by MEW, SLA or 2PP prior to, or after volumetric bioprinting. Furthermore, the photopolymerizable resin could be modified with another process prior to volumetric bioprinting to create discrete structures or gradients within the printed construct.

### 2.7 Increasing Complexity of Living Building Blocks—The Potential for Fabrication with Organoids

Organoids are stem cell derived self‐organized mini‐tissues which have certain morphological and functional characteristics of natural human organs.^[^ ^111^ ^]^ They differ from spheroids in that organoids are formed by natural growth and differentiation (self‐organization), while spheroids are fabricated by artificial (bio)assembly from cell suspensions.^[^ ^112^, ^113^ ^]^ There are already at least four published papers which suggested (but not implemented yet) the combination of bioprinting^[^ ^112^, ^114^ ^]^ and biofabrication^[^ ^3^, ^115^ ^]^ with rapidly emerging organoid technologies. Organoids usually have irregular shape, they are not vascularized and do not have stroma and innervation, but they have internal histological structure closely resembling the histomorphology of natural human organs. Moreover, they recapitulate certain physiological functions of human organs. In this context, human organoids are more advanced and potentially more attractive alternative to tissue spheroids in the development of hybrid biofabrication technologies. Modern biofabrication and microfluidics technologies can enable vascularization and innervation of organoids and, thus, could made them physiologically and morphologically even more relevant and similar to native human tissue and organs.^[^ ^116^ ^]^ In particular, the main future development will be the automated 3D manufacture and increased complexity and throughout offered by hybrid biofabrication and organoid technologies that offer significant breakthroughs in developmental biology, drug and disease screening in precision medicine.^[^ ^3^ ^]^ Engineering of organoids of desirable size and shape is already under investigation using specially designed biofabrication technologies and novel biopolymers and hydrogels.^[^ ^112^, ^113^, ^117^ ^]^ There is a role for biomaterials in organoid research,^[^ ^118^ ^]^ and in establishing fabrication methods to investigate/control their behavior.^[^ ^117^, ^119^ ^]^ Sizing, shaping, and directed differentiation of organoids using advanced biomaterials is already started^[^ ^112^, ^113^ ^]^ and could be developed further using advanced hybrid fabrication technologies.

## 3 Implications of Future Hybrid Fabrication Technologies

The use of hybrid fabrication in this review is directed toward TERM, biofabrication and bioprinting. In the context of hybrid fabrication, there are several implications for these fields. First, a hybrid approach enables the development of more sophisticated and more biomimetic TERM scaffolds and tissue constructs. Second, the hybrid approach is able to increase scaffold porosity without compromising its biomechanical properties and viability. Third, hybrid biofabrication can increase the initial cell density and, thus, save time otherwise necessary for cell proliferation and tissue engineered construct maturation. Finally, both manual and robotic‐based hybrid technologies allow greater precision assembly of cell and tissue spheroids.

### 3.1 Increasing the Available Volume for Tissue Constructs

Some of the most recent contributions by MEW involve being able to reinforce a matrix while maintaining an overall low volume fraction. We believe that this is essential for voluminous tissue constructs that require both cell‐friendly matrices and handling capabilities. So far, there have been two general directions for TE; scaffold‐based and scaffold‐free.^[^ ^77^ ^]^ One can argue that there is a middle‐ground of “high porosity” scaffolds, including those made via MEW, that have a low polymer volume fraction than most other scaffolding technologies.^[^ ^120^ ^]^ In these cases, the volume occupied by a non‐cell‐penetrating structure is less than 10%, and currently as low as 2%.^[^ ^92^ ^]^ The substantial mechanical reinforcing effects observed by well‐positioned, low‐micrometer dimension fibers provides another argument for such “high porosity” approaches. In a different context, increasing the overall volume of TERM constructs to the centimeter scales required for human tissue has been a long‐standing challenge in the field.^[^ ^7^ ^]^ Such an overall volume of tissue constructs or high cell densities require a vascularization strategy depending on their intended use,^[^ ^28^, ^116^ ^]^ well reviewed elsewhere.^[^ ^121^ ^]^

### 3.2 Increasing Complexity of Scaffolds and Tissue Constructs with Biomimicry

Hybrid fabrication strategies offers practically unlimited opportunities for creative combination of different technologies in order to achieve highly desirable histotypical organization of tissue constructs with maximal possible level of complexity and authenticity. Closely packed tissue spheroids can, after tissue fusion, reach a higher density than in natural tissue and organs. In certain tissues (for example, mesenchymal condensation in cartilage during embryonic chondrogenesis) this increased high density is a necessary precondition for sequential initiation of extracellular matrix synthesis and deposition.^[^ ^69^ ^]^ Thus, it is expected that combining tissue spheroids with high packing cell density will provide more voluminous TERM products. In turn, vascularization strategies become important to maintain nutrition.

This leads toward the several levels of complexity within natural tissue and organs that require addressing. The establishment of a complex vasculature, chemotactic gradients, heterogeneous cell densities biomechanical cues and mimicking the ECM distributions are just some areas where current fabrication technologies are far away from matching the delicate and complex distribution for natural tissue. Previous hybrid fabrication research has touched upon this, including the integrated tissue–organ printer that combines melt extrusion and bioprinting for the fabrication of various vascularized musculoskeletal tissue.^[^ ^7^ ^]^ Mechanical reinforcement principles, as previously outlined, have been first performed with melt extrusion and bioprinting/spheroid printing for bone/cartilage tissues^[^ ^20^, ^52^ ^]^ and recently followed by MEW for cartilage and heart valve applications.^[^ ^42^, ^98^ ^]^ Approaches to vascularize tissues by Kolesky et al., also showed how combining different extrusion based multimaterial bioinks and fugitive inks can be used for a more complex vascularized structure for liver biofabrication,^[^ ^28^ ^]^ and for more large volume tissues.^[^ ^122^ ^]^ In another important study on complex vasculature fabrication, the DLP of a hydrogel allowed the mechanical environment of vascular channels surrounding alveolar to be studied.^[^ ^6^ ^]^ More recently, the potential of a multimaterial switching during bioprinting was demonstrated that could further increase distributions requiring a change in cell density and mechanical reinforcement.^[^ ^123^ ^]^ Cell self‐organization can be somewhat relied upon to establish such biomimicry, the improved resolution of the “fabrication technology toolbox” to establish a sufficient microenvironment for tissue maturation is likely required. While replicating the complexity of natural tissue may seem insurmountable to perform with current fabrication technologies, improved resolutions with new fabrication technologies and their hybridization with others could advance our capability in this aspect.

Improvements in fabrication resolution may also converge with our improved ability to map the cellular and molecular distributions within tissue. A separately developing field is the process of tissue clearing^[^ ^124^ ^]^ which uses protocols to render entire organs/tissues such as the brain, spinal cord, eye and kidney, transparent. Combining tissue clearing with immunohistochemistry and light‐sheet microscopy, a fully 3D mapping of organs at the cellular and even molecular level can be obtained. There are several tissue‐clearing protocols such as CLARITY,^[^ ^125^ ^]^ 3DISCO^[^ ^126^ ^]^ and, more recently, SHANEL^[^ ^127^ ^]^ that can provide greater volumetric information at the cellular level on intact organs. We foresee that such tissue clearing processes will converge with biofabrication through the digitization of discrete tissue at this cellular and volumetric level. Such information will converge with improved manufacturing resolutions to provide an increasingly complex biomimetic structure for printing pathways. Developing volumetric models of the 3D distribution of cells and ECM within an organ will become increasingly important for biofabrication. Interestingly, deep learning and neural networks have been applied to the analysis and understanding of such treated tissues. Excellent research articles^[^ ^125^, ^126^, ^127^ ^]^ describing in‐depth such tissue clearing methods and reviews^[^ ^128^, ^129^ ^]^ can be found elsewhere.

### 3.3 Manual or Automated—More Precision Placing of Cells and Spheroids

When combining cells or cell‐laden matrices to AM scaffolds, manual pipetting remains the most commonly used approach, often related to cost. For example, in a breakthrough paper, Laronda et al. used manual pipetting to place tissue spheroids (ovarian follicles) onto 3D printed gelatin constructs (Figure 4C).^[^ ^72^ ^]^ This implantation of an artificial printed “ovary” into sterilized mice to restore their fertile function provided a dramatic demonstration of how biofabrication can tackle significant medical challenges.^[^ ^72^ ^]^ This trend of automation is even progressing toward in situ bioprinting^[^ ^130^ ^]^ of cartilage,^[^ ^131^ ^]^ skin,^[^ ^132^ ^]^ and cranial defects.^[^ ^133^, ^134^ ^]^

The variance of results and the increasing complexity of experiments demands that such processes eventually require automation. This transition is becoming economically easier with more options for low‐cost automated dispensing technologies becoming available. Since the central hypothesis of biofabrication is that hierarchical cell/spheroid placement is essential to recapitulate more in vivo like tissue constructs, then defined dispensing and direct writing is an inevitable but important evolution in this aspect.

Biofabrication, and the accompanying automation could alter the long‐appreciated challenge of primary cell efficacy such as a loss of potency, change in phenotype and factors associated with rapid cell proliferation. Automation won't remove the issue of loss of phenotype but the repeatable cell handling steps can reduce variation make the most of potency available. Integration of fabrication systems with bioreactor systems may also provide opportunity for tissue maturation in this sense.^[^ ^3^ ^]^ Automated fabrication strategies that do not require cells but deliver cues or factors that can recruit host cells or modulate repair offer new opportunities to potentially circumvent this loss in primary cell efficiency.

Another manual dispensing example involves fiber‐reinforced hydrogels that have been primarily made by manual dispensing of precursors prior to gelling. Such improvements in mechanical properties are also observed with hybrid MEW and 3D bioprinting approaches. The need for more reproducible outcomes and more discrete hierarchical structures within biofabrication will drive the replacement of manual dispensing with automated systems. The fluid mechanics and variations currently observed with the seeding of cells is supporting the hypothesis that adopting a fully automated dispensing system will improve both the quality and reproducibility of experiments.

### 3.4 Assembly Lines and Multifunctional Biofabrication Printers

The design and implementation of automated biofabrication devices is another important trend. It opens a direct pathway for automated, scalable, standardized and cost‐effective biofabrication of complex tissue constructs which is critically important for successful clinical translation and commercialization of TERM products.

**Figure 8:**
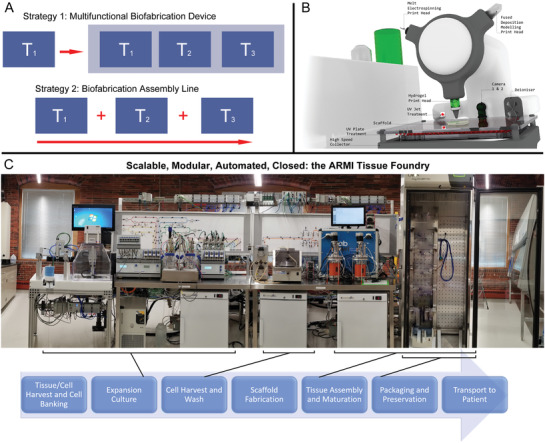
Different strategies in the development of hybrid fabrication technologies (T1, T2, T3). A) Schematic demonstration of two different strategies for the hybridization of different manufacturing technologies: strategy 1 based on construction of integrated fabrication assembly line consisted of separate biofabrication devices (T1+T2+T3) and strategy 2 based on combination of different biofabrication technologies integrated into one single multifunctional device (T1/T2/T3). B) A practical implementation of strategy 1: a multifunctional printer concept incorporating several types of heads that allow hybrid fabrication. C) A practical example of implementation of strategy 2 as a biofabrication assembly line developed to automate tissue construct preparation. (B) is previously unpublished and was kindly provided by Dr. Felix Wunner and Professor Dietmar Hutmacher. (C) is previously unpublished and was kindly provided by Tom Bollenbach, Ph.D., ARMI | BioFabUSA.

Another strategic question is how hybrid fabrication technologies will be implemented together. Based on previous approaches this would be through i) the use of a multifunctional device combining a single set of different functional capabilities or ii) by development of separated mono‐functional devices integrated into one united biofabrication assembly line^[^ ^135^ ^]^ (**Figure** **8**A). Moreover, certain fabrication technologies are not possible to integrate into one device due to certain technological restraints and limits.

There is also the availability of multifunctional biofabrication printers that contains all of the separate technologies to be hybridized, and maintain it in a single location.^[^ ^74^ ^]^ There are several commercial multifunctional biofabrication printers based around the technology of extrusion‐bioprinting (Figure 8B). With such multifunctional printers, the tissue construct is fabricated in a single location, and the different technologies combine at this point. In fact many commercial 3D bioprinters have thermoplastic extrusion heads on them, so as to perform hybrid fabrication and, in turn, will drive further research on this topic.

Currently there are a number of commercial and laboratory‐based multifunctional biofabrication machines that allow for hybrid construct fabrication for tissue engineering and regenerative medicine and has been recently reviewed.^[^ ^136^ ^]^ In general these machines utilize a collection of rapidly interchangeable print head, each offering a specific function and control systems such and temperature, pressure, flow rate etc. Fabrication technologies of each print head can typically include the following:

1. Extrusion hydrogel bioprinting of cell laden bioinks.
2. Extrusion thermoplastic polymer dispensing.
3. MEW of thermoplastic polymers with associated collector.
4. Melt or solution electrospinning with associated collector.
5. Piezo or jet‐based bioprinting (or inkjet printing).
6. Fluidic or needle (Kenzan) based bioassembly of spheroids.
7. Microfluidic print head containing multiple nozzles for multimaterial/multibioink dispensing.

There are several initiatives that reduce human interactions from cell culture procedures, such as the “Skin Factory” biofabrication assembly line (now disassembled) developed by Fraunhofer IGB and other modular, assembly line biofabrication systems.^[^ ^33^ ^]^ More recently, a low‐cost and modular, assembly line approach is being taken at the Advanced Regenerative Manufacturing Institute (ARMI), in Manchester, USA. This is an enclosed system built outside a cleanroom, which saves on costs and accessibility. With the backing of industrial partners, the purpose of ARMI and its BioFabUSA program is to develop the engineering tools for automated tissue construction. The first of many planned automated assembly line prototypes (Figure 8C) has been built to identify improvements for the next version. Ultimately, factory‐line systems should be reduced in size to a similar footprint of a multifunctional printer. In the context of the numerous diseases and injuries to organs/tissue, full automation is ultimately the level of control necessary to deliver a scalable product that is both safe and validated.

## 4 Conclusions

The evolving field of TERM, and more recently biofabrication, will be further advanced by the hybridization of manufacturing processes. There are already several variants of hybrid technologies which have been developed based on the combination of at least two fabrication technologies, and further combinations should accelerate in the next five years. Different technologies within a biofabrication or TERM approach must complement each other and provide better outcomes through synergistic effects. In this review, recent fabrication technologies—MEW, volumetric bioprinting, Kenzan method and spheroid formation—have been illustrated as examples of technologies where the full potential remains to be investigated. Two future strategies of hybrid manufacturing, either in multifunctional devices or with a fabrication assembly line have been discussed and outlined.

## Conflict of Interest

The authors declare no conflict of interest.
